# Spectrum of lytic lesions of the skull: a pictorial essay

**DOI:** 10.1007/s13244-018-0653-y

**Published:** 2018-09-19

**Authors:** Lorenzo Ugga, Renato Cuocolo, Sirio Cocozza, Andrea Ponsiglione, Arnaldo Stanzione, Vito Chianca, Alessandra D’Amico, Arturo Brunetti, Massimo Imbriaco

**Affiliations:** Department of Advanced Biomedical Sciences, University of Naples “Federico II”, Via Pansini, 5, 80131 Naples, Italy

**Keywords:** Review, Skull, Diagnostic imaging, Neoplasms, Radiologists

## Abstract

**Abstract:**

Lytic lesions of the skull include a wide range of diseases, ranging from benign conditions such as arachnoid granulations or vascular lacunae, to aggressive malignant lesions such as lymphomas or metastases. An early and correct characterisation of the nature of the lesion is, therefore, crucial, in order to achieve a fast and appropriate treatment option. In this review, we present the radiological appearance of the most frequent lytic lesions of the skull, describing findings from different imaging modalities (plain X-rays, CT and MRI), with particular attention to diagnostic clues and differential diagnoses.

**Teaching Points:**

• *Osteolytic skull lesions may be challenging to diagnose.*

• *Association of different imaging techniques may aid image interpretation.*

• *Clinical information and extensive knowledge of possible differential diagnoses is essential.*

• *Some osteolytic tumours, although benign, may present as locally aggressive lesions.*

• *Malignant lesions require accurate staging, followed by variable treatment approaches.*

## Introduction

Intradiploic skull vault lesions are rare, often incidental findings in diagnostic imaging. These lesions may also be found during staging examinations or investigating local clinical symptoms. Their interpretation requires the evaluation of several pieces of information, among which are imaging characteristics and clinical setting. Osteolytic lesions usually pose greater diagnostic dilemmas, as they share many imaging features.

Skull X-ray is usually the first diagnostic tool on which lesions are identified, but its use is decreasing due to the availability of more accurate imaging techniques, such as computed tomography (CT) and magnetic resonance imaging (MRI).

CT is useful for the evaluation of lesion density (sclerotic or osteolytic), extension assessment (focal or diffuse) and recognition of possible pathognomonic patterns, such as the trabecular pattern of haemangioma. Furthermore, it is the best modality for the depiction of possible periosteal reaction. A better tissue characterisation and evaluation of bone marrow involvement is obtainable on MRI. It is also more accurate than CT in the assessment of intra- and extracranial extension.

In this review article, we will discuss pseudolesions (e.g. venous lacunae, arachnoid granulations), benign and malignant osteolytic lesions, focusing on those that most frequently occur in clinical practice.

## Pseudolesions

Arachnoid granulations (AGs) are cerebrospinal fluid (CSF)-filled protrusions of the arachnoid in the dura mater. Protruding into the brain’s dural sinuses, they allow for CSF resorption. AGs are frequently found in parasagittal areas, along the superior sagittal and transverse sinuses, near the confluence of a cortical vein into the sinus. They show a high prevalence in the healthy population, being reported in approximately two-thirds of individuals. Usually, their size ranges from 2 to 8 mm, but, sometimes, the maximum diameter can exceed 10 mm [1]. In such cases, they are referred to as “giant AGs”, proceeding in differential diagnosis with other dural entities, such as extradural masses and venous thrombosis [2].

**Fig. 1:**
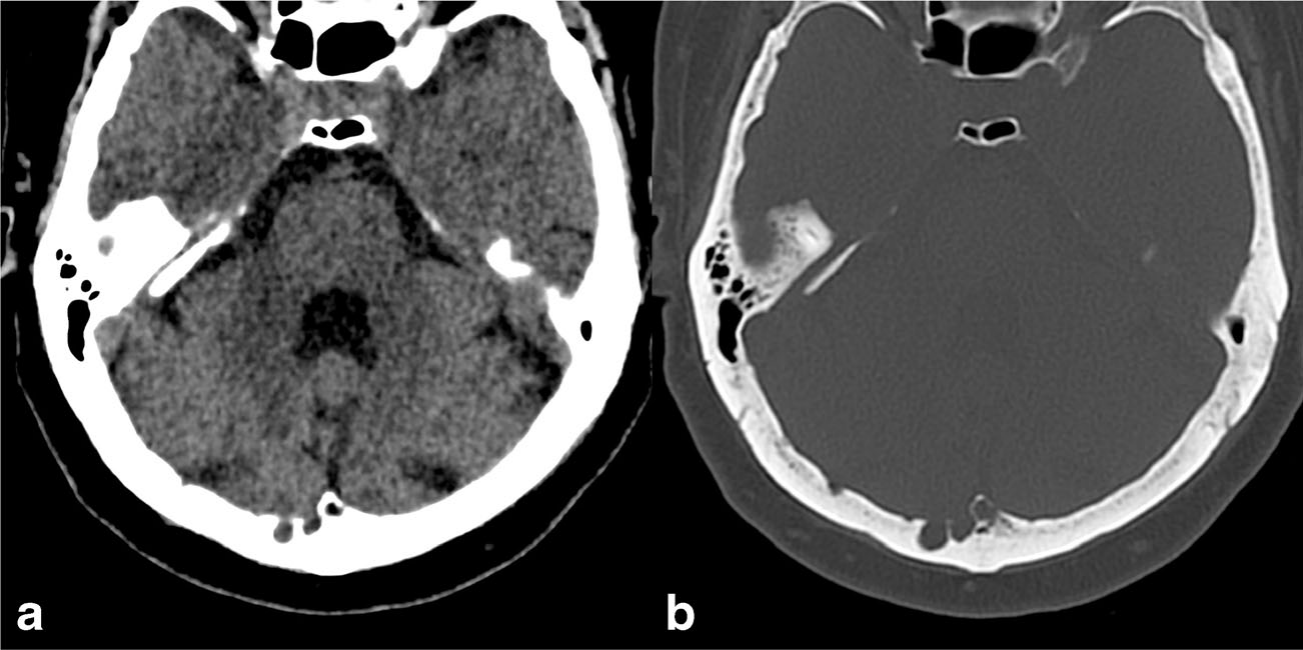
Arachnoid granulations (AGs). Axial computed tomography (CT) images with brain (**a**) and bone (**b**) windows showing two well-defined, round-shaped lesions with cerebrospinal fluid (CSF) density, protruding into the calvarium along the right transverse sinus

Due to their content, on CT scans, AGs appear as well-defined, round-shaped lesions with CSF density, protruding into the calvarium, causing a filling defect (Fig. 1). Similarly, on MR images, AGs are characterised by low signal on T1-weighted images and hyperintensity on T2-weighted images, hypointense on fluid-attenuated inversion recovery (FLAIR) sequence and without contrast enhancement. However, “giant AGs” may show atypical MRI findings, with higher-than-expected T1- and FLAIR-weighted signal [2].

**Fig. 2:**
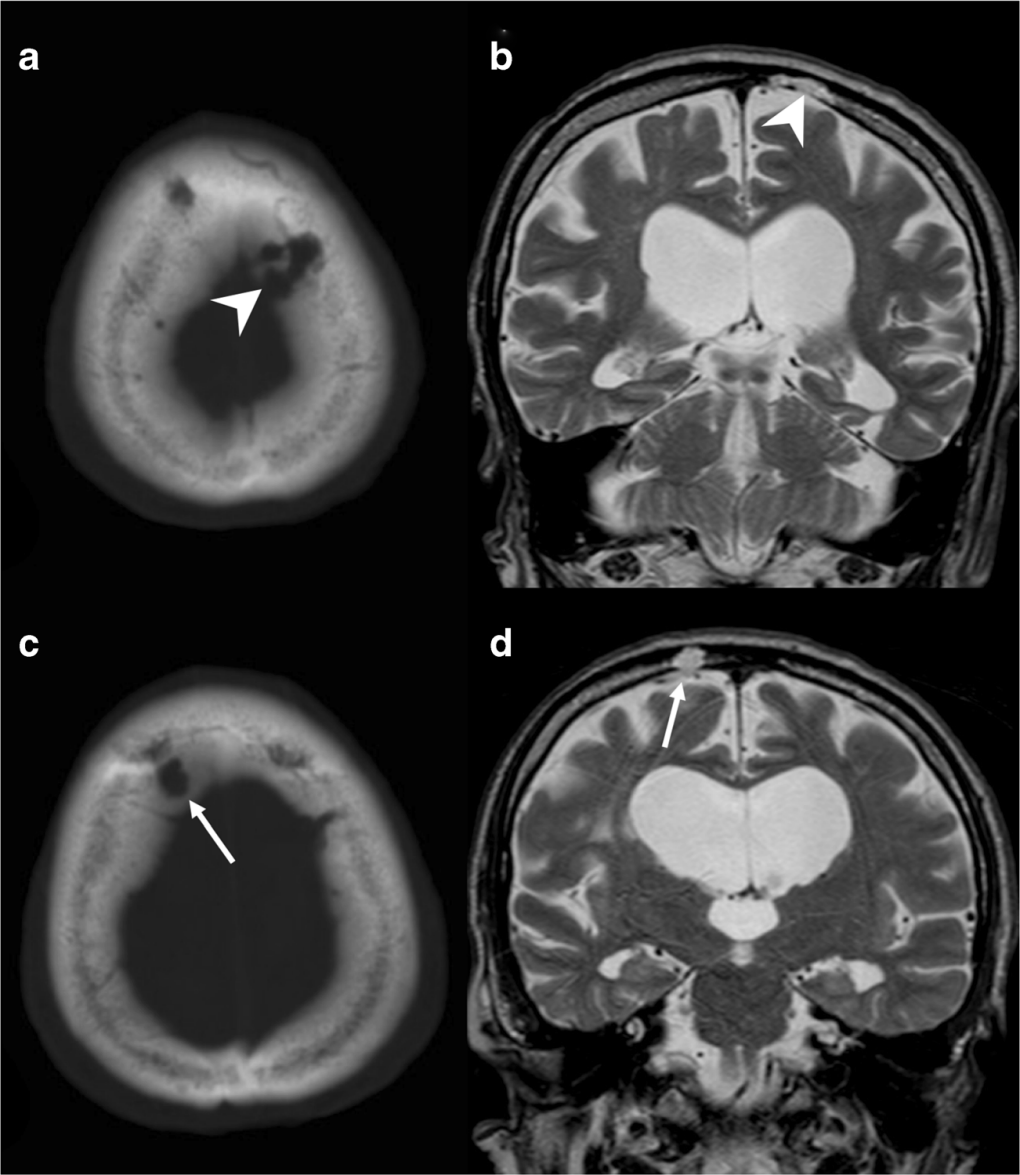
Venous lacuna (VL). Axial CT image with bone (**a**) window showing a lytic lesion within the left parasagittal diploic space (*arrowhead*), confirmed on T2-weighted sequence (**b**). In a caudal slice, a right parasagittal AG can be depicted (**c**, **d**)

Venous lacunae (VL) consist of enlarged venous spaces within the parasagittal dura, adjacent to the superior sagittal sinus [3]. Usually, three VL are localised in each hemisphere, at the frontal, parietal (the most common location) and occipital lobes. They receive blood from superficial cortical and meningeal veins, and CSF from AG. Some VL can enlarge, extending from the skull inner table into the diploic space, providing a lytic image of the skull on CT scan (Fig. 2). They behave as pure vascular entities, with contrast enhancement on both CT and MRI.

## Epidermoid and dermoid cysts

Lesions with inclusion of ectopic tissue consist of epidermoid and dermoid cysts (ECs and DCs, respectively), differing in complexity [4]. Several theories have been proposed to explain their development: they may derive from ectodermal inclusions during neural tube closure from the third to fifth weeks of embryogenesis or be acquired as a result of trauma or surgery. An intradiploic location is less common compared to other intracranial locations [5].

Intradiploic epidermoid cysts (IECs) are the most common, occurring at any age from the first to seventh decades of life, with no gender prevalence. They are preferentially found in the frontal, parietal and occipital regions, often affecting multiple bones [6].

IECs, due to their slow growth, usually show a small diameter at diagnosis [7]. However, larger lesions have been reported, presenting with signs of intracranial hypertension and focal neurological signs. IECs are benign, with infrequent malignant transformation, which may occur especially in cases of recurrence due to incomplete resection.

On plain X-rays, IECs may present as rounded or lobulated lytic areas with smooth and sclerotic margins.

**Fig. 3:**
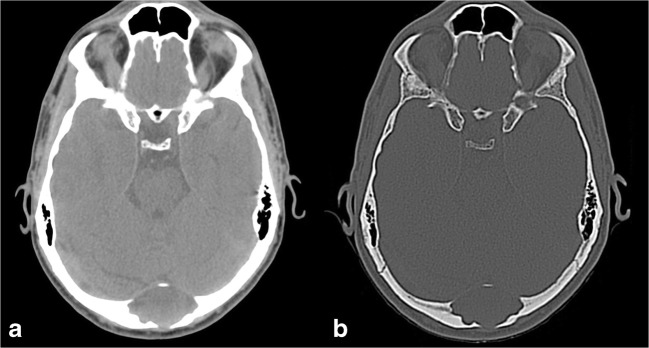
Intradiploic epidermoid cyst (IEC). Axial CT images with soft tissues (**a**) and bone (**b**) windows show an occipital hypodense osteolytic lesion with smooth margins and greater involvement of the inner table, extensively demineralised

At CT scans, non-enhancing hypodense focal lesions are their typical presentation, with associated sharply demarcated bony defects and calcifications, and the inner table generally more affected than the outer (Fig. 3).

**Fig. 4:**
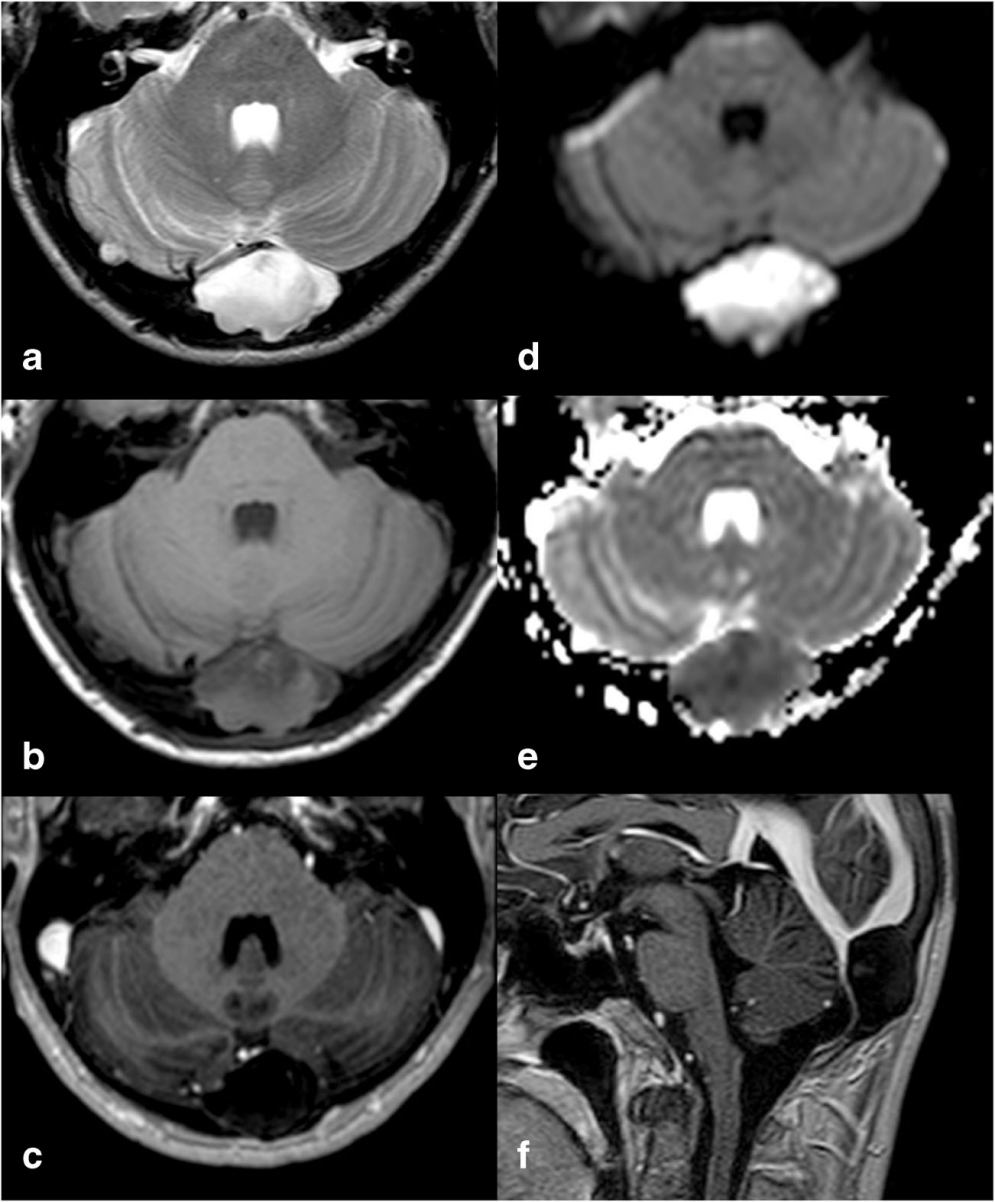
IEC. On MR images of the same patient shown in Fig. 3, the lesion appears slightly inhomogeneous, mostly hyperintense on T2w (**a**) and hypointense on T1w (**b**) images, without contrast enhancement (**c**), showing diffusion restriction on *b* 1000 diffusion-weighted imaging (DWI) and the apparent diffusion coefficient (ADC) map (**d**, **e**). It causes compression on the confluence of sinuses (sagittal contrast-enhanced T1w image, **f**). As a consequence, the patient presented with intracranial hypertension symptoms

MR images show the presence of a mildly T1-hyperintense lesion, with iso-/hyperintensity on both T2-weighted and FLAIR images, restricted diffusion on diffusion-weighted imaging (DWI) and no contrast enhancement [8] (Fig. 4).

DCs are lined by stratified squamous epithelium but, unlike ECs, contain epidermal appendages, such as hair follicles, sweat glands and sebaceous glands. These lesions, frequently occurring in childhood, with the highest incidence during the first or second decades of life, show a slow growth and tend to develop on the midline along the course of suture lines, near the anterior fontanelle [9, 10]. Their appearance ranges from simple localised cysts to complex lesions, with dermal sinus and possible intracranial extension [11].

Histological differences with IECs may reflect in different imaging findings [12]. While plain X-ray shows the presence of a focal lucency, hard to distinguish from IECs, CT may allow a differential diagnosis by the demonstration of fatty content. Their CT appearance ranges from well-defined, homogeneous and low-attenuating lobulated masses to highly inhomogeneous due to a combination of saponification, microcalcification and blood products.

Finally, MRI depicts a strong and spontaneous T1-hyperintensity, again related to its lipid contents, coupled to variable signal on T2-weighted images and without significant changes after contrast administration, although a peripheral rim of enhancement may be observed [13].

## Intraosseous haemangiomas

Intraosseous haemangiomas (IH) are benign vascular tumours characterised by slow or absent growth. The skull is their second most frequent localisation after the spine, with frontal and parietal bones being the more often affected sites. IH show their prevalence peak during the fourth and fifth decades of life, with females being more often affected than males [14].

Most skull IH are cavernous lesions, containing dilated blood vessels separated by fibrous septa. They are generally asymptomatic but can manifest as painful, palpable masses.

Plain X-rays usually show a well-circumscribed, round or oval osteolytic lesion, with a classic “spoke-wheel” appearance.

**Fig. 5:**
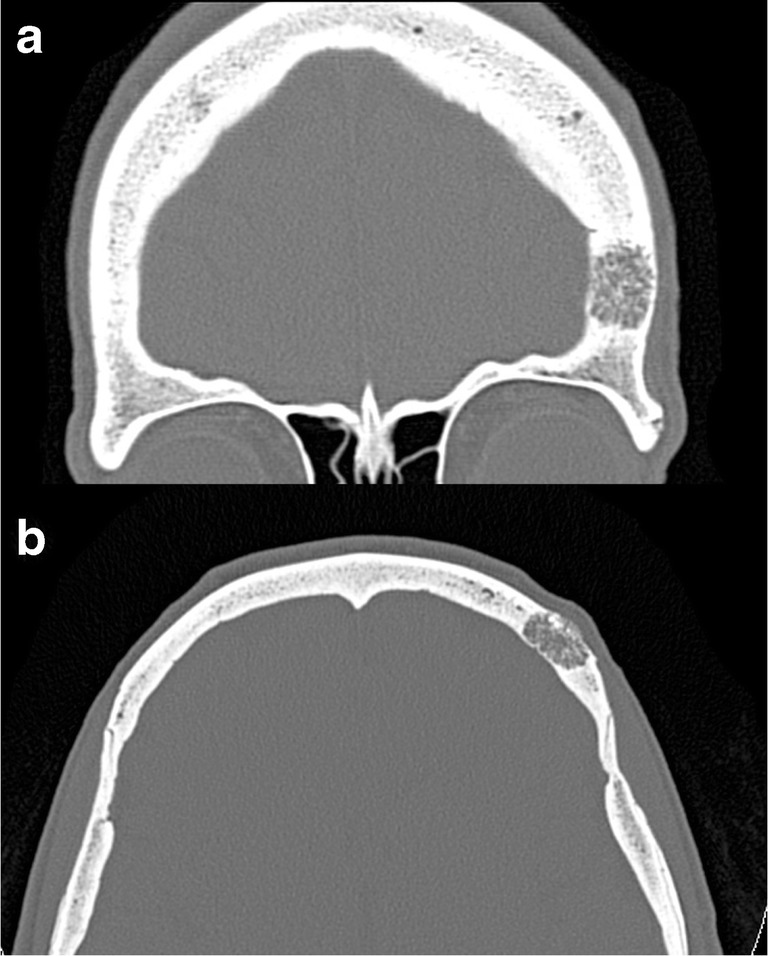
Intraosseous haemangioma (IH). Coronal (**a**) and axial (**b**) reformats of a CT scan with bone window indicate a left frontal expansive lesion with thin margins and intralesional spicules, radiating from a common centre, which erodes the outer layer of the skull, with relative sparing of the inner table

On CT scans, they appear as expansive lesions with thin margins and intralesional spicules, radiating from a common centre, with marked and homogenous enhancement after contrast administration. They generally erode the outer layer of the skull, with a relative sparing of the inner table [15] (Fig. 5).

MR images show the presence of lesions with variable signal intensity, related to the amount of fat content. Usually, IH appear as isointense lesions on T1-weighted images, with possible scattered hyperintense foci due to fat components.

T2-weighted images show a hyperintense lesion with multiple lobules, representing the vascular lacunae.

Finally, post-gadolinium T1-weighted sequences depict, similarly to CT scans, a vascular lesion with strong homogenous enhancement, better delineated on fat-saturated images.

In case of complications, such as neurological symptoms due to mass effect or haemorrhages, excision is indicated, while embolisation can be useful before surgery. If surgical removal is not feasible, radiotherapy can be considered as an alternative treatment option.

## Aneurysmal bone cyst

Aneurysmal bone cysts (ABCs) are benign, cystic lesions composed of multiple blood-filled cavities divided by connective tissue septa, rarely affecting the skull (2–6% of all cases).

Local swelling and tenderness represent the most common clinical findings. Pulsation can be observed if the lesion breaks through the inner table of the cranium.

In most cases, an association with other primary benign or malignant bone lesions is present. These include giant cell tumours, fibrous dysplasia, non-ossifying fibromas, IH, osteoblastomas, simple bone cysts, chondroblastomas, chondromyxoid fibromas and osteosarcomas [16].

**Fig. 6:**
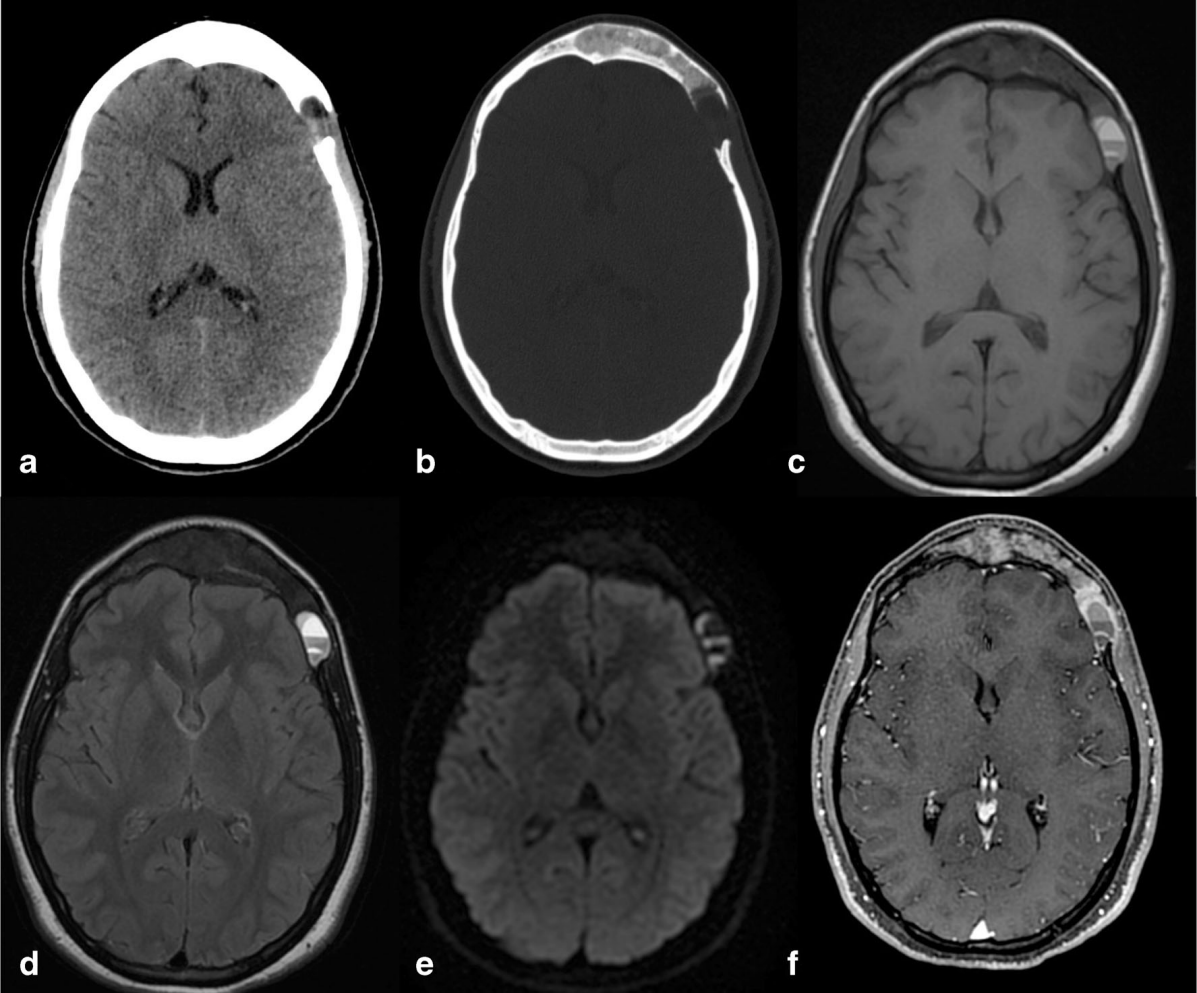
Aneurysmal bone cyst (ABC). Axial CT images with brain (**a**) and bone (**b**) windows show a left frontal lytic lesion with blood-fluid levels, associated with a diffuse ground-glass modification of the frontal bone, related to the presence of a fibrous dysplasia. Magnetic resonance (MR) examination better depicts the multiloculated lesion, confirming blood-fluid levels on T1w (**c**), FLAIR (**d**) and DWI (**e**) images, and showing enhancement of the capsule and internal septa (**f**)

At the level of the skull, it often occurs as a secondary phenomenon in pre-existing fibrous dysplasia (Fig. 6).

The imaging hallmark of an ABC is the presence of an expansive osteolytic lesion with a narrow transition zone and blood-fluid levels due to the layering of blood products. However, these findings are not specific, as they have also been reported in other conditions, such as telangiectatic osteosarcoma, malignant fibrous histiocytoma, fibrous dysplasia, synovial sarcoma, haemangioma and simple bone cyst [17].

On CT scans, ABCs appear as well-demarcated, multiloculated, osteolytic lesions, with blood-fluid levels. MRI is the most appropriate imaging technique, depicting additional information, such as the presence of internal septa and a better, more accurate lesion definition and representation of blood-fluid levels with identification of blood in different stages [18].

The ideal treatment for ABCs is surgical excision. If such an approach cannot be performed, radiation therapy is warranted, although with consideration of the high recurrence rate of the disease.

## Bone desmoplastic fibroma

Bone desmoplastic fibroma (DF) is a rare, intraosseous and locally aggressive neoplasm, accounting for less than 0.1% of all primary bone tumours [19].

On pathology, it appears as a lesion mainly constituted by spindle cells, with abundant collagen component and dilated vascular channels. It can infiltrate surrounding soft tissues, although conserving its benign characteristics, without showing necrosis, cellular atypia or mitotic activity [20].

DFs frequently affect long bones and mandible, with skull involvement being reported in a very low percentage of cases [21].

When occurring at this level, they usually present as slow-growing mass lesions, possibly associated with headache, hearing changes, ear drainage and head asymmetry [22].

**Fig. 7:**
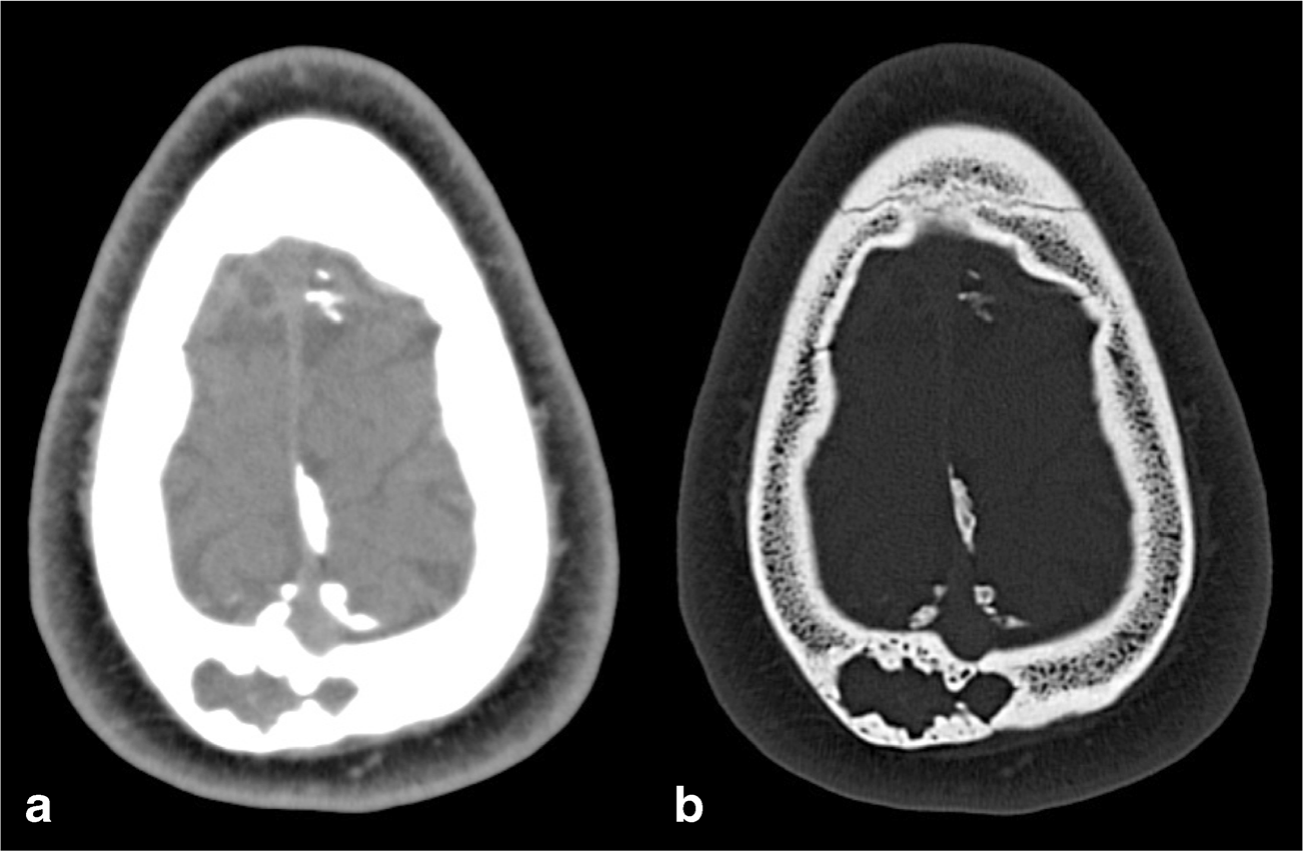
Bone desmoplastic fibroma (DF). Axial CT images with soft tissues (**a**) and bone (**b**) windows showing a right parietal lytic lesion without erosion of the inner and outer tables

On CT scans, DFs are typically represented as lytic lesions with thinning of both inner and outer tables of the skull (Fig. 7), while on MRI, these lesions present with heterogeneous signal intensity on T2-weighted images and a low degree of enhancement after contrast administration, due to the presence of dense connective tissue and hypocellularity.

**Fig. 8:**
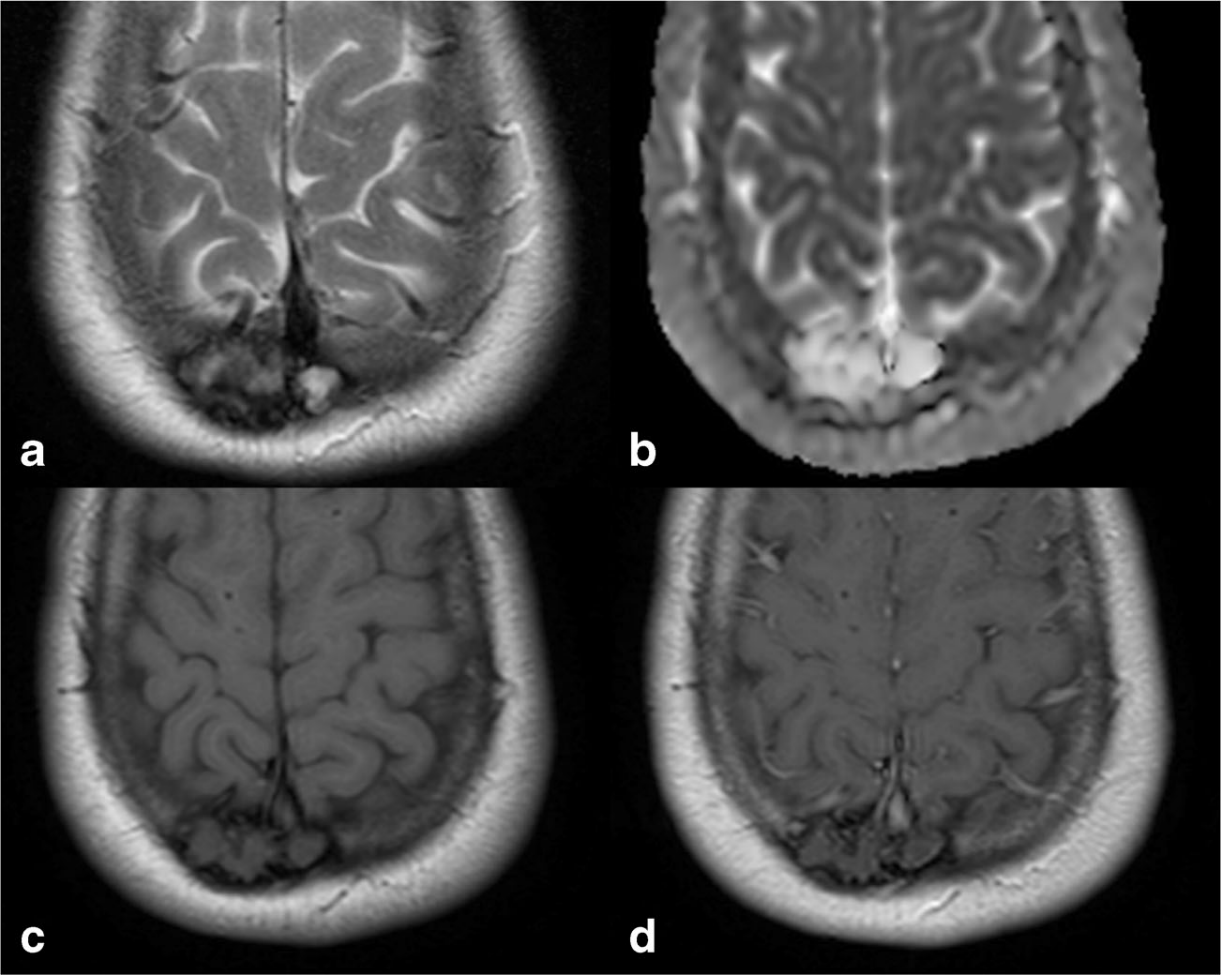
DF, same patient shown in Fig. 7. On MRI, the lesion presents a typical low signal intensity on T2w and T1w sequences (**a**, **c**), without diffusion restriction on the ADC map (**b**) or enhancement after contrast administration (**d**)

These imaging findings are not peculiar of DFs, being present in other conditions, such as fibrous dysplasia, haemangioma, eosinophilic granuloma, low-grade osteosarcoma or metastasis. Nevertheless, significant T2-weighted shortening of a non-sclerotic fibro-osseous lesion should recall DFs in the differential diagnosis of lytic skull lesions [23] (Fig. 8).

Due to the frequent local recurrence rate, wide total surgical resection is the treatment of choice for DFs.

## Eosinophilic granuloma

Eosinophilic granuloma (EG) is the benign form of Langerhans cell histiocytosis (LCH), a multisystemic rare disorder of unknown aetiology, mainly affecting male children, characterised by the proliferation of Langerhans cells [24]. EG usually presents as a single skeletal lesion, often involving flat bones, with the skull being affected in more than 50% of the cases [25]. EG is characterised by fever, localised pain, soft tissue swelling and mild peripheral eosinophilia, and it usually shows a benign clinical course, also considering its high sensitivity to radiation therapy [26].

On plain radiographs, EG usually appears as a well-defined round or ovoid lytic lesion, with non-sclerotic margins and a “punched out” presentation, that rarely extends across cranial sutures or determine transdural or intra-axial invasion. Due to their lytic nature, lesions could contain a small sequestrum of devascularised bone, surrounded by lucency, providing a typical “bull’s eye” appearance. Furthermore, if the inner cranial table is more involved than the outer, the lesion could result in a double contour image (“hole within a hole” sign). Finally, if multiple lesions are present, they can enlarge and merge, providing a geographic skull appearance.

**Fig. 9:**
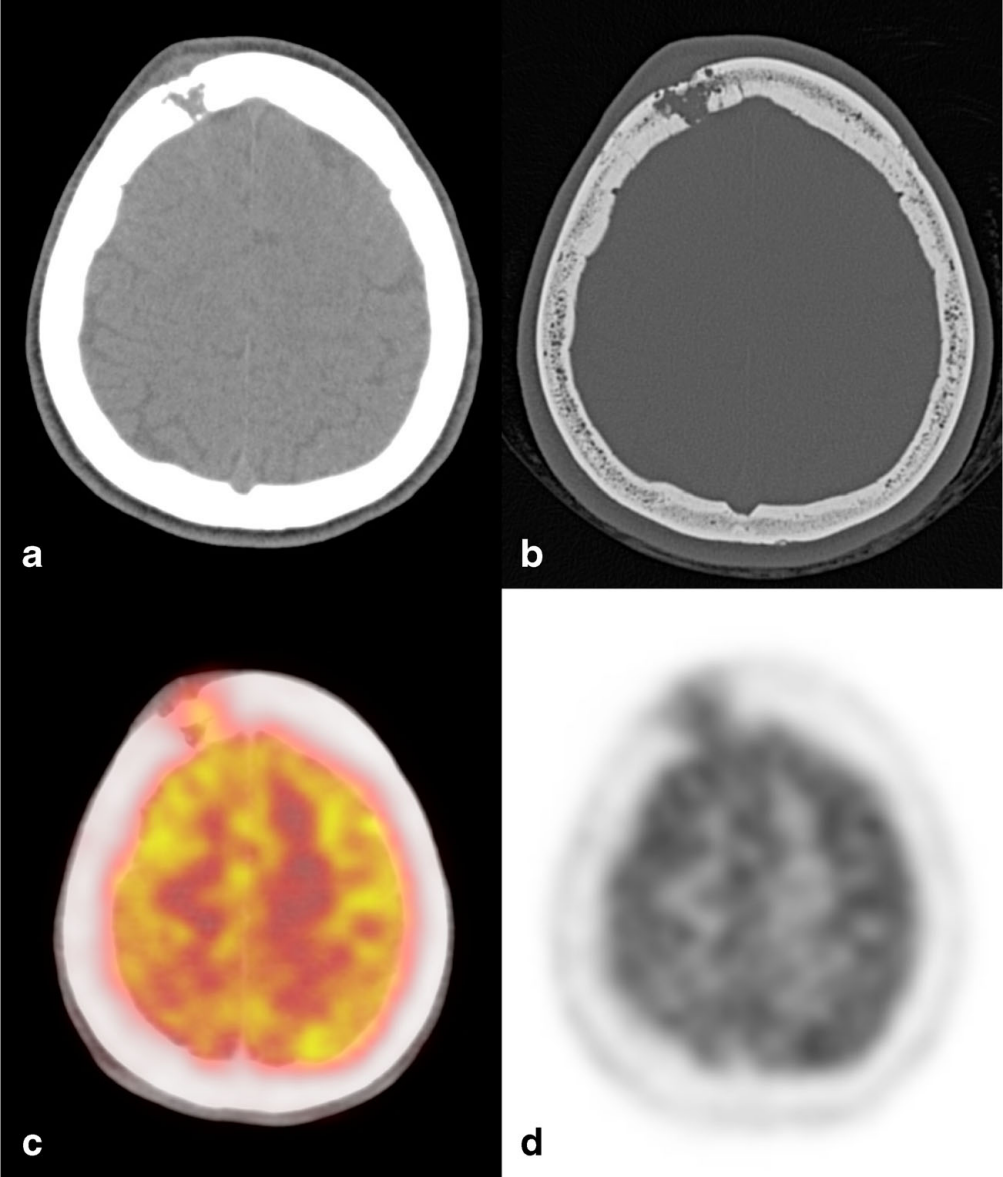
Eosinophilic granuloma (EG). Axial CT images with soft tissues (**a**) and bone (**b**) windows showing a right frontal lytic lesion with ill-defined margins extending in the contiguous extracranial soft tissues. Erosion of the inner cranial table is more pronounced than of the outer (“hole within a hole” sign). 18(F) FDG-PET CT examination demonstrates a moderate tracer uptake (**c**, **d**)

CT findings are similar to those evaluable on plain X-rays, providing an excellent evaluation of bone erosions, as well as a good quantification of the involvement of the surrounding soft tissues. CT images are usually acquired for surgical planning purposes, as well as to perform targeted biopsies in equivocal case presentations [27, 28] (Fig. 9).

**Fig. 10:**
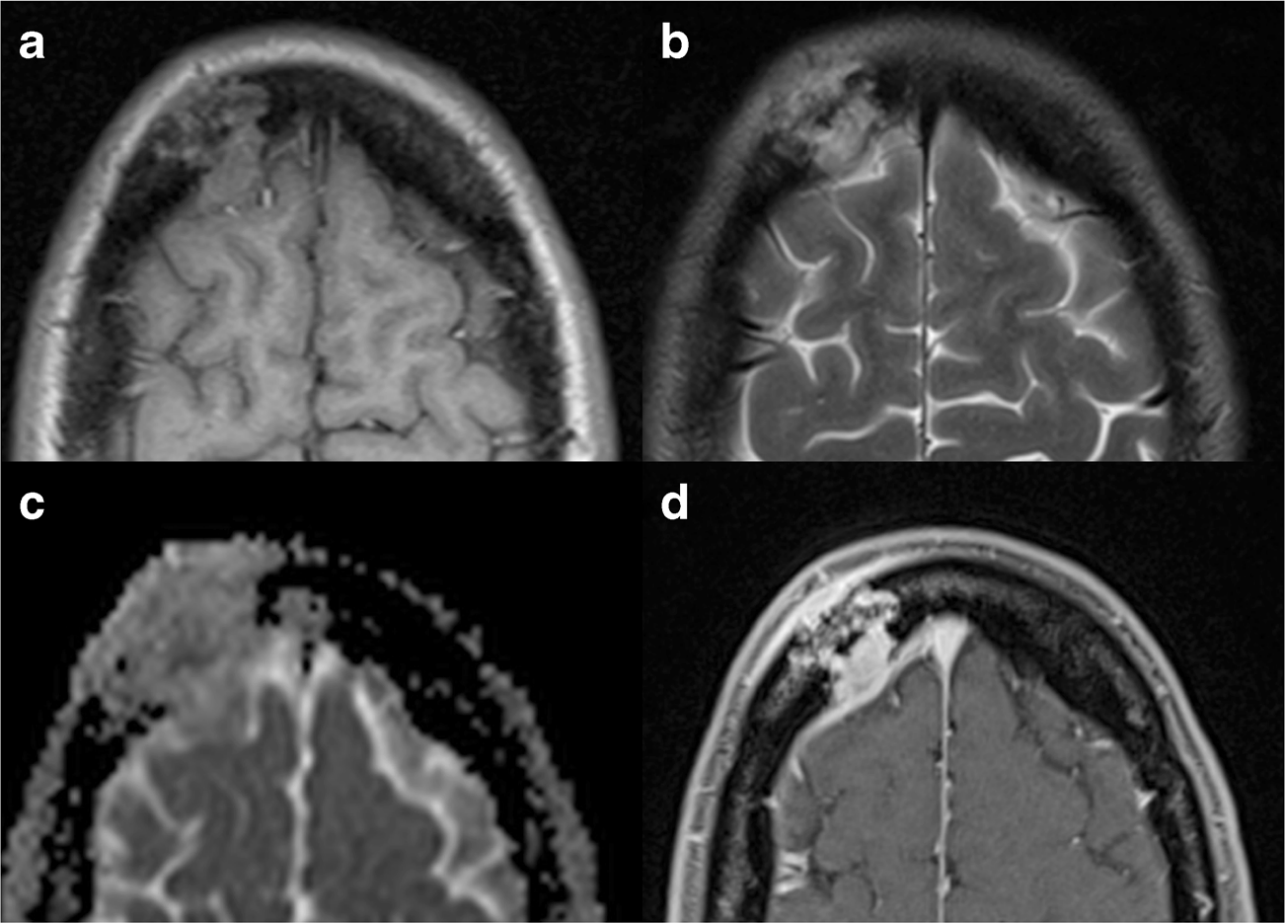
EG, same patient shown in Fig. 9. On T1w (**a**) and T2w (**b**) images, the lesion appears mildly inhomogeneous, with diffusion restriction on the ADC map (**c**). MRI allows for an accurate evaluation of the surrounding soft tissues, as well as intracranial extension after contrast administration (**d**), causing a moderate dural thickening

MRI is usually performed to obtain an accurate evaluation of the surrounding soft tissue changes, as well as the medullary extent of the lesion [29]. EG usually appears as isointense to hypointense on T1-weighted images, slightly hyperintense on T2-weighted images, with this hyperintensity more pronounced on short-inversion-time inversion recovery (STIR) images, and is frequently characterised by contrast enhancement after gadolinium administration (Fig. 10).

## Lymphoma

Primary lymphoma of the bone is a rare neoplasm composed of malignant lymphoid cells. It represents 7% of all malignant bone tumours, with involvement of flat bones, especially the skull, being infrequent [30]. It may occur in a single skeletal section or in multiple segments, with or without visceral or nodal extension. Osseous lymphoma belongs to the non-Hodgkin lymphoma classification, being specifically a diffuse large cell lymphoma [31].

The usual clinical presentation is the presence of scalp swelling, without neurological deficits. If intraparenchymal infiltration is present, neurological deficits may become the most important clinical manifestation of disease [32].

The radiological appearance of primary bone lymphoma is highly variable and not specific [33]. It ranges from nearly normal-appearing bone, to an extensive permeative process with cortical and soft-tissue involvement. Nonetheless, some characteristic patterns that can aid in the differential diagnosis from other lytic skull lesions can be identified.

**Fig. 11:**
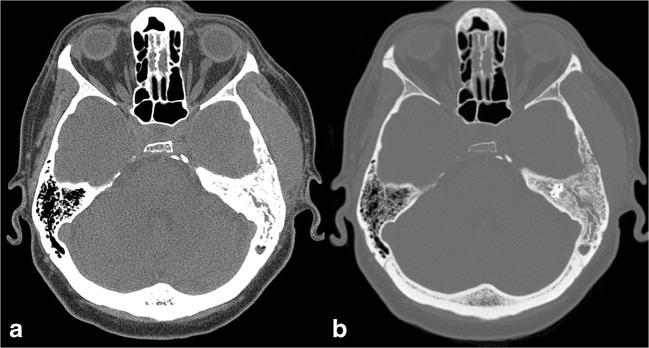
Primary lymphoma of the bone. Axial CT images with soft tissues (**a**) and bone (**b**) windows highlight the presence of a bone lesion of the squamous portion of the left temporal bone, with lytic permeative pattern, extending to temporal extracranial soft tissues

The most common radiological feature of primary lymphoma is the occurrence of a lytic to destructive lesion with permeative or moth-eaten appearance (Fig. 11), while the presence of focal lytic areas with well-circumscribed margins is less frequent [31].

**Fig. 12:**
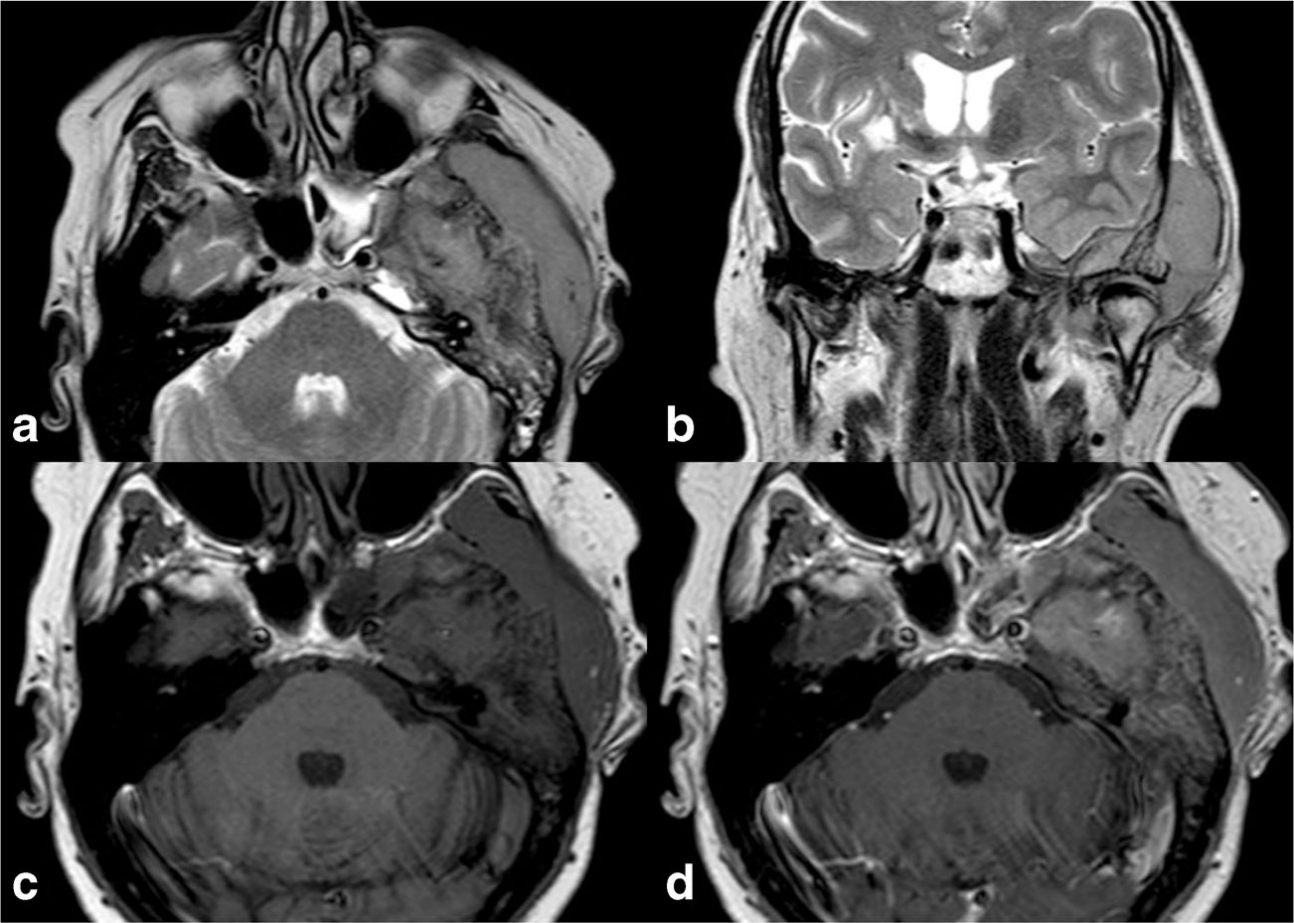
Primary lymphoma of the bone, same patient shown in Fig. 11. On MRI, the lesion demonstrates low T2 signal on axial (**a**) and coronal (**b**) T2-weighted sequences and extension to the temporal fossa and to the epidural space of the middle cranial fossa. The tumour exhibits homogeneous contrast enhancement; axial T1-weighted before (**c**) and after (**d**) contrast administration

In the case of lesions with unclear radiographic and CT findings, MRI is a valuable tool to achieve a radiological diagnosis. Indeed, T1-weighted imaging is able to distinguish between normal fatty marrow and infiltrative lesions, with the latter showing a lower signal intensity. On the other hand, lesions show a higher signal intensity on T2-weighted images compared to the normal bone marrow, especially when peri-tumoural oedema is present. However, if fibrotic components are present, a low T2-weighted signal can be present. The use of fat-suppressing techniques, such as STIR, may better delineate lesions from the surrounding fatty bone marrow [34]. Finally, MRI is able to provide a good visualisation of soft-tissue involvement, which is very frequent and can be present even in the absence of visible cortical alterations (defining the above-mentioned permeative pattern of the disease) [35] (Fig. 12).

The treatment of primary bone lymphoma is still controversial, and various radiation therapy approaches have been proposed, with or without associated chemotherapy.

## Multiple myeloma

Among primary malignant bone neoplasms affecting adults, multiple myeloma (MM) is the most common, accounting for around 1% of all new cancer cases [36]. MM results from monoclonal proliferation of malignant plasma cells, which release immunoglobulins and infiltrate haemopoietic locations.

The distribution of MM lesions reproduces those physiologically observed in the red marrow, with the axial skeleton showing the highest prevalence of involvement [37].

**Fig. 13:**
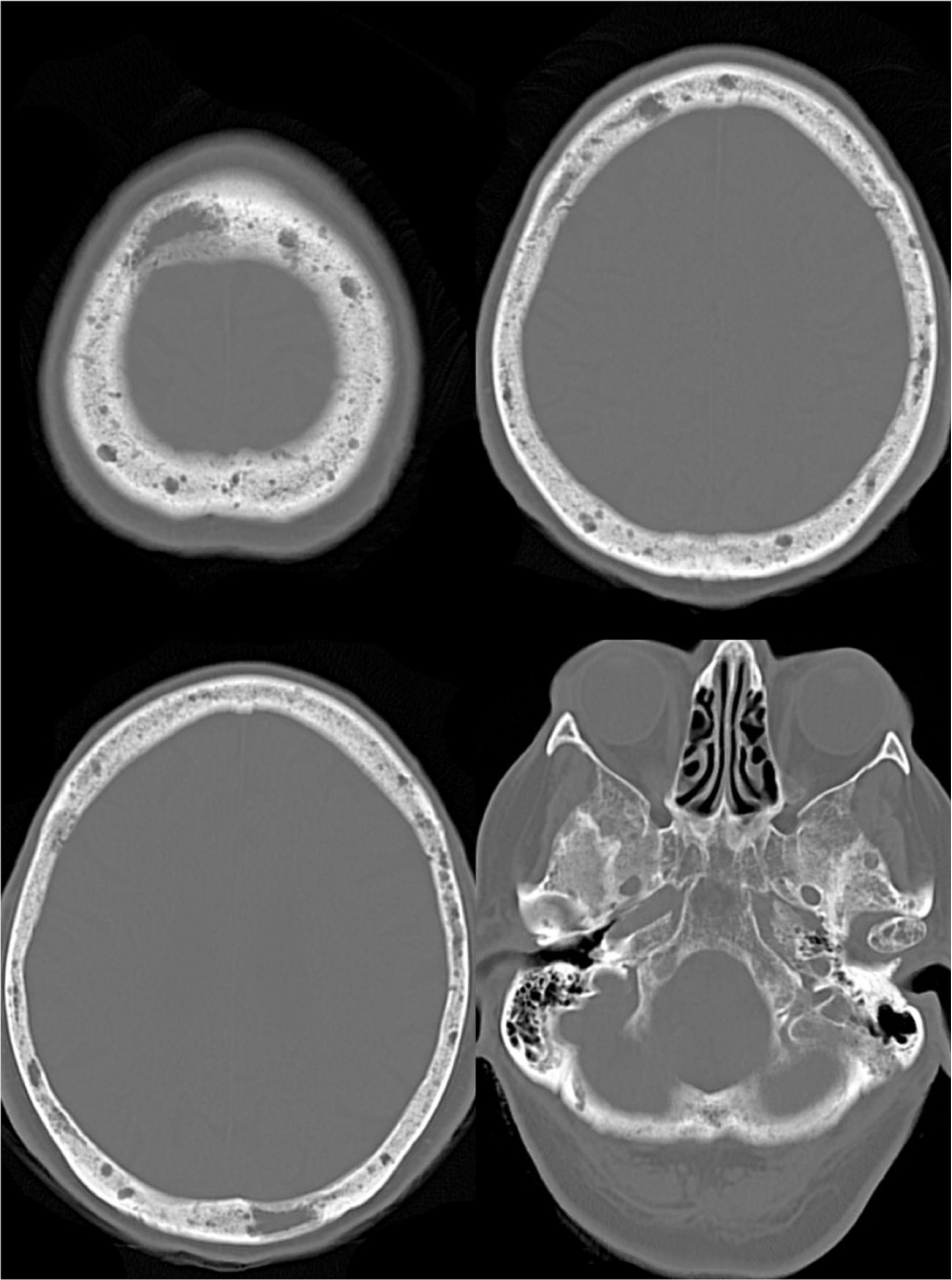
Multiple myeloma (MM). Axial CT images with bone window showing multiple osteolytic lesions varying in shape and size, involving the skull vault and base

When the skull is affected, its most common radiological presentation is represented on both plain radiograms and CT scans by numerous and well-circumscribed lytic bone lesions (Fig. 13). To better evaluate the bone marrow infiltration, MRI is usually performed, also providing high sensitivity in the detection, even in the absence of lysis [38, 39].

## Metastases

Skull metastases follow a complex series of events, dependent on the characteristics of the primary malignancy. This process involves multiple signalling pathways and molecular interactions, and an extensive and growing list of factors has been reported [16]. These characteristics are the principal element to determine their lytic or blastic behaviour [40, 41]. Usually, when malignant cells reach the bone marrow niche, they stimulate bone resorption, explaining why bone metastases are often lytic in appearance. Notable exceptions are prostate and breast cancers, where the osteoblastic reaction is more prominent [42]. Bone metastases are asymptomatic in most cases. However, if clinical manifestations are present, pain is frequently the most common presenting symptom.

Osteolytic metastases of the skull usually present as abnormal radiolucencies in the calvarial bones. Lesions are often multiple, with well-defined margins, with diffuse infiltration of the skull more rarely encountered [15]. They can extend in the contiguous soft tissues, sometimes with a permeative growth pattern characterised by the presence of multiple small endosteal areas of resorption with a relative sparing of the skull cortex. While lytic metastases are not generally specific to the site of origin of the primary lesion, the presence of a blowout pattern may aid in the differential diagnosis. In these cases, renal cell and thyroid carcinoma are the most commonly responsible, even if this pattern may be seen in other carcinomas, such as breast or lung cancers [16].

On MRI scans, skull metastases usually present as lesions involving the diploe and the cortical bone, with T1-weighted hypo- to isointense signal and T2-weighted hyperintensities. Post-contrast appearance is highly variable, depending also on the primary tumour pathology. Indeed, lesions show different behaviours, ranging from marked and homogeneous to no enhancement at all, with some others showing scattered or rim-like hyperintensities [43].

**Fig. 14:**
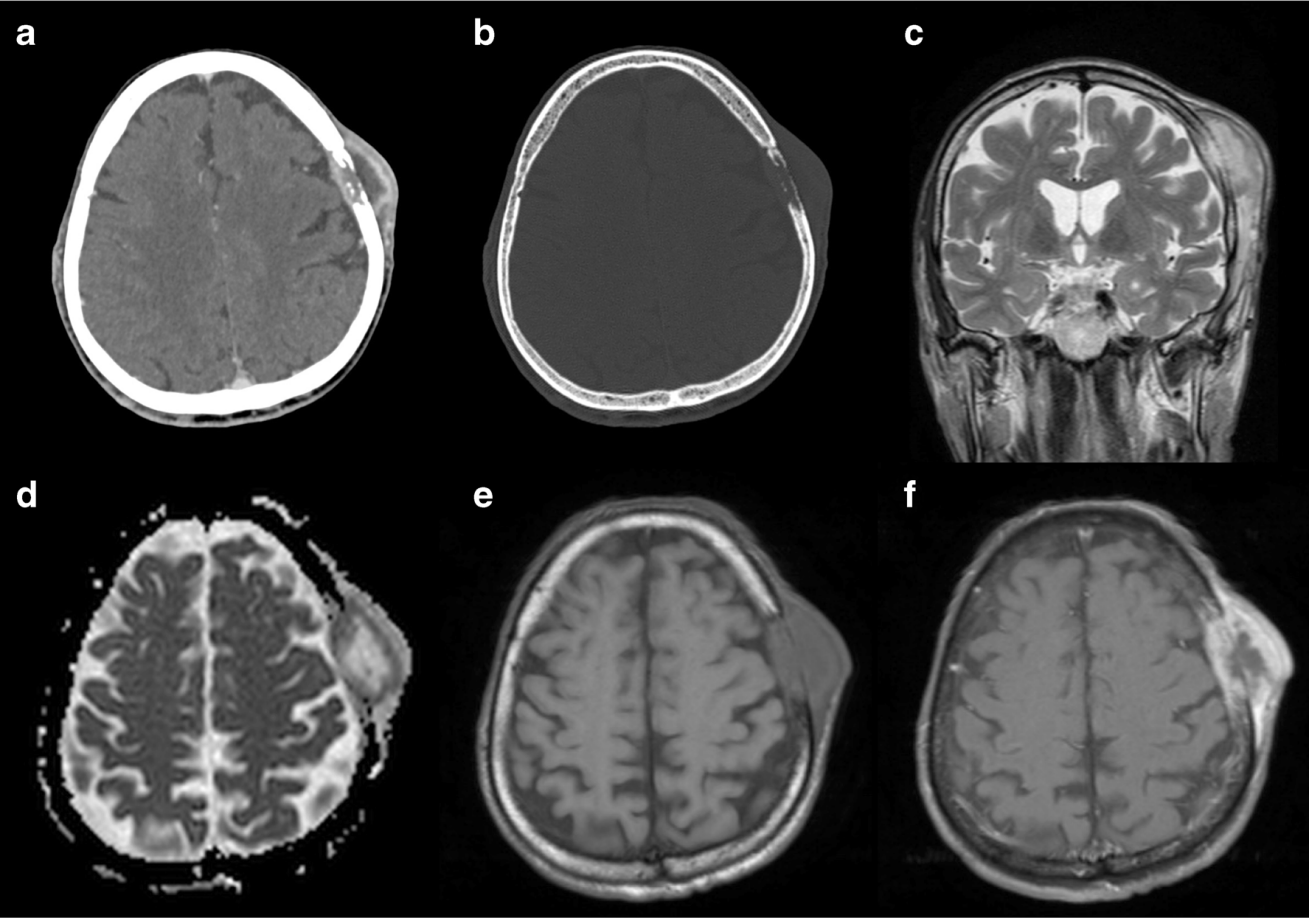
Metastasis. Axial contrast-enhanced CT, with brain (**a**) and bone (**b**) windows showing a left parietal lytic, widely necrotic, lesion with intra- and extracranial extension. MRI better depicts extraosseous spread on both intra- and extracranial aspects, with dural displacement and thickening, better seen on coronal T2w image (**c**). The lesion presents diffusion restriction (**d**) and enhancement (**e**, **f**) of the peripheral solid component. T1w (**e**) and T1w after contrast administration with fat saturation (**f**) images

Furthermore, MRI is crucial in the evaluation of the involvement of surrounding and soft tissues, especially of the dura mater (dural tail sign), other than other associated intraparenchymal lesions (Fig. 14).

Similarly to imaging findings, the nature of the primitive cancer determines the treatment options. External radiotherapy represents the current treatment option in adjunction to the systemic chemotherapy [44]. A surgical approach is not usually indicated, with its role limited to the treatment of possible complications (e.g. haemorrhages, mass effect etc.).

## Conclusion

Osteolytic calvarial lesions are rare findings, possibly challenging from a diagnostic viewpoint. In this paper, we have presented their respective essential imaging findings. Summarising, there are some semeiotics that can help the radiologist in the differential diagnosis. First of all, there are highly suggestive findings that we wish to highlight, such as: a focal intradiploic filling defect with involvement of the inner cortex in typical locations is characteristic of pseudolesions; the presence of fluid–fluid levels in an intradiploic cystic lesion is suggestive of aneurysmal bone cyst (ABC); a trabecular structure is indicative of haemangioma. When these patterns are not recognisable, other signs can help distinguish benign and malignant lesions. Secondly, erosion of the cortex suggests a malignant lesion, while its expansion suggests a benign one. Periosteal reaction also mainly occurs in locally aggressive lesions, as do irregular margins and epidural or soft tissue extracranial extension. Finally, multiple lesions are more frequent in some malignant conditions, such as multiple myeloma (MM) and metastases. Diffusion restriction on diffusion-weighted imaging (DWI) may help identify highly cellular malignant lesions, although it must not be forgotten that not all malignant lesions show this pattern, while some benign lesions, in particular epidermoid cysts, also show restriction.

In conclusion, in the evaluation of osteolytic calvarial lesions, different imaging techniques, especially computed tomography (CT) and magnetic resonance imaging (MRI), are useful in differentiating benign from malignant lesions. In difficult cases, their interpretation may be aided by the association of clinical information and extensive knowledge of possible differential diagnoses and pseudolesions.

## Abbreviations

ABCs: Aneurysmal bone cysts
AG: Arachnoid granulations
CSF: Cerebrospinal fluid
DCs: Dermoid cysts
DF: Bone desmoplastic fibroma
ECs: Epidermoid cysts
EG: Eosinophilic granuloma
FLAIR: Fluid-attenuated inversion recovery
IECs: Intradiploic epidermoid cysts
IH: Intraosseous haemangiomas
LCH: Langerhans cell histiocytosis
MM: Multiple myeloma
VL: Venous lacunae
